# How do GP practices and patient characteristics influence the prescription of antidepressants? A cross-sectional study

**DOI:** 10.1186/s12991-015-0041-7

**Published:** 2015-01-22

**Authors:** Alain Mercier, Jacques Benichou, Isabelle Auger-Aubin, Jean-Pierre Lebeau, Estelle Houivet, Paul Van Royen, Lieve Peremans

**Affiliations:** Department of General Practice, Rouen University, CIC Inserm 0204, 1 rue de Germont, 76031 Rouen Cedex, France; Department of Biostatistics, Inserm U 657, University of Rouen, Rouen University Hospital, 1 rue de Germont, 76031 Rouen Cedex, France; Department of General Practice, Département de médecine générale, Université Paris Diderot, Sorbonne Paris Cité, Paris, France; EA Recherche clinique coordonnée ville-hôpital, Méthodologies et Société (REMES), 75010 Paris, France; Department of General Practice, Tours University, 10, Boulevard Tonnellé, B.P. 3223, 37032 Tours Cedex 1, France; Department of Primary and Interdisciplinary Care, Faculty of Medicine, Health Science University of Antwerp, Antwerp, Belgium; Department of Public Health, Vrije Universiteit Brussel, Brussels, Belgium; Department of General Practice, University Paris 13, Sorbonne Paris Cité, Bobigny, France; Department of Family practice, Faculty of Medicine, Rouen University, 20 Bd Gambetta, 76000 Rouen, France

**Keywords:** Antidepressants, General practice, General practitioners, Cross-sectional study, Pharmaco-epidemiology

## Abstract

**Background:**

Under-prescription of antidepressants (ADs) among people meeting the criteria for major depressive episodes and excessive prescription in less symptomatic patients have been reported. The reasons influencing general practitioners’ (GPs) prescription of ADs remain little explored. This study aimed at assessing the influence of GP and patient characteristics on AD prescription.

**Methods:**

This cross-sectional study was based on a sample of 816 GPs working within the main health care insurance system in the Seine-Maritime district of France during 2010. Only GPs meeting the criteria for full-time GP practice were included. The ratio of AD prescription to overall prescription volume, a relative measure of AD prescription level, was calculated for each GP, using the defined daily dose (DDD) concept. Associations of this AD prescription ratio with GPs’ age, gender, practice location, number of years of practice, number of days of sickness certificates prescribed, number of home visits and consultations, number and mean age of registered patients, mean patient income, and number of patients with a chronic condition were assessed using univariate and multivariate analysis.

**Results:**

The high prescribers were middle-aged (40–59) urban GPs, with a moderate number of consultations and fewer low-income and chronic patients. GPs’ workload (e.g., volume of prescribed drug reimbursement and number of consultations) had no influence on the AD prescription ratio. GPs with more patients with risk factors for depression prescribed fewer ADs, however, which could suggest the medications were under-prescribed among the at-risk population.

**Conclusions:**

Our study described a profile of the typical higher AD prescriber that did not include heavy workload. In future work, a more detailed assessment of all biopsychosocial components of the consultation and other influences on GP behavior such as prior training would be useful to explain AD prescription in GP’s practice.

**Electronic supplementary material:**

The online version of this article (doi:10.1186/s12991-015-0041-7) contains supplementary material, which is available to authorized users.

## Background

Antidepressants (ADs) are increasingly prescribed and used worldwide, especially in all industrialized countries. In the USA, Medicaid programs’ spending on antidepressants increased from $159 million in 1991 to $2 billion in 2005 [1] and reached $9.6 billion in 2008 [2]. AD prescription in France increased sixfold, from € 84 million to € 525 million, between 1980 and 2005 [3]. This high AD prescription level, mainly coming from primary care, is a source of concern. With the exception of use for severe major depressive episodes, there is scarce evidence on the effectiveness of AD use by physicians in primary care [4]. The question of whether their prescription is appropriate or not is a subject of debate. Some data showed excessive prescription in patients with symptoms not meeting all diagnostic criteria, yet misdiagnosis can lead to under-prescription among truly depressed people [5,6]. Many reports support the finding of an increasing rate of non-psychiatric AD prescriptions [7-10]. The growing evidence regarding the usefulness of AD prescription for non-psychiatric conditions could also explain this apparently inadequate prescription rate [11,12]. Though high psychotropic drug consumption in some countries such as France could be related to higher prevalence of depression or to overall high drug consumption, the available data do not allow reliable comparisons between countries [13,14].

There is no consensus on the appropriateness of AD prescription by general practitioners (GPs). The criteria for defining “an appropriate prescription” for a given indication differ among authors, and their assessment is prone to variations in measurement, heavily influencing findings on assessment of the rate of inadequate prescriptions. Across all countries, GPs are responsible for 80% of AD prescriptions [15]. AD prescription rates and habits vary greatly among GPs [16]. “High” prescribers might deal with patients having more comorbidities and more risk factors for depression, such as age, the presence of a chronic disease, and a low income level, but the findings on these points are not consistent [9,17]. GPs’ prescription of ADs might also be influenced by their demographic characteristics (e.g., age, gender, and place of practice) or profile of care (e.g., overall prescription volume and number of patients in their practice) [18]. The aim of this cross-sectional study was therefore to analyze the influence of patient characteristics and GP demographic characteristics and care profile on AD prescription based on data from GP practices in France in 2010.

## Methods

### Study population

This cross-sectional study was based on the analysis of characteristics and prescription data of a GP sample from the Seine-Maritime district in northwestern France during the year 2010. In 2010, this area had a population of 1,248,443 inhabitants and had 1,040 registered GPs. Recorded data from the main local health insurance system (CNAM-TS) were used. All data on drug prescriptions and characteristics of physicians and patients are routinely collected in this database each time a patient asks for prescribed drug reimbursement. Collected data are thus rather exhaustive and reliable.

**Figure 1:**
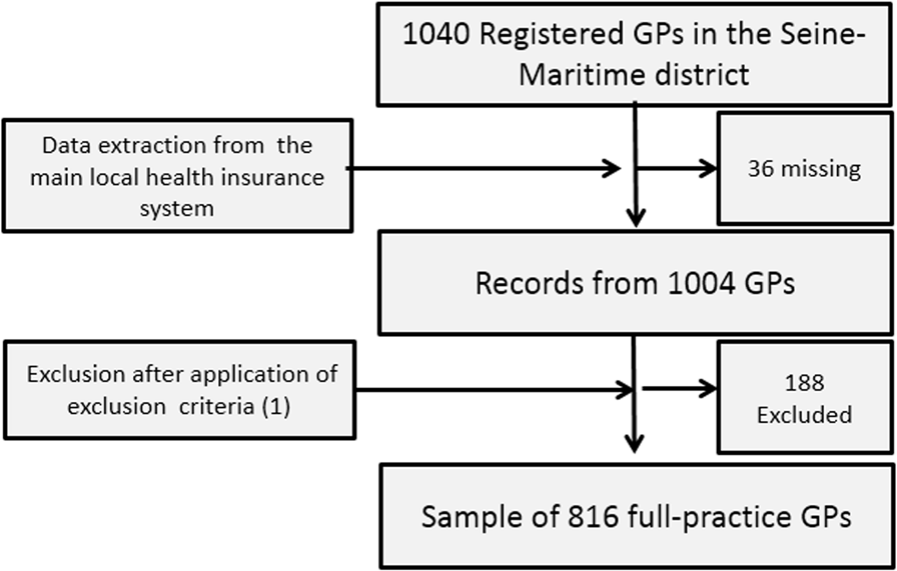
**Flow chart of data extraction.** Among the physicians recorded as GPs, 17 were recorded with an exclusive particular mode of practice (e.g. acupuncture), 13 did not prescribe any sick leave, 24 had no chronic patients, 42 had no low-income patients, 4 had no conventional agreement with the health care system, 156 performed less than one consultation per day, and 16 had less than two registered patients.

Among the 1,046 registered GPs, data from 36 GPs were missing, thus leaving data for 1,004 registered GPs for further selection. As our aim was to analyze data coming from full-time standard primary GP practice, the following exclusion criteria were applied to the initial data sample of physicians registered as GPs. “Specialized” physicians with a predominant particular mode of practice (PMP) such as osteopathy or acupuncture, as well as those practicing homeopathy or nutrition exclusively, were excluded. Other exclusion criteria were prescription of no or only a few medications, no sickness certificates prescribed, performing no home visits, and having only a part-time GP activity (e.g., a GP practice combined with hospital or emergency department practices). GPs performing less than one reimbursed consultation per day, and having no contractual agreement with the French national health insurance system, were also excluded. GPs were also excluded when they did not treat chronic patients (i.e., no patients with any of the 30 chronic diseases allowing patients to be treated free of charge in France) or any very low-income patients (i.e., no patients who can see a GP free of charge based on their very low-income level as defined by French law). After applying these criteria, the data for 816 GPs were retained and constituted the analysis sample. The different steps of data selection are presented in Figure 1.

Agreement of the French National Commission for Data Protection (Commission Nationale de l’Informatique et des Libertés) was obtained (Decision DE 2010 097).

### Data

The first set of variables described GP’s practice, i.e., the GP’s age and gender, practice location (urban or rural based on the postal code and on the National Institute of Statistics and Economic Studies’ (INSEE) classification), the year when they began practicing medicine, and the part-time or full-time nature of their work. The second set of variables described medical activity during the entire year of 2010, i.e., the number of sick leave days prescribed per year, the number of home visits and consultations, the number of registered patients, the mean patient age, the number of very low-income patients (“free of charge” patients), and the number of patients with a registered chronic condition. The final set of variables described the prescription activity for the entire year of 2010, i.e., the overall number of prescription orders, number of units of drug prescribed for all active ingredients, as a whole, and detailed data for each AD molecule (The list of ADs included is available in Additional file 1).

### Assessment of the AD prescription ratio

As there is no universally recognized measure of AD prescription level, an AD prescription ratio was defined using the following steps. For each GP, yearly prescription volumes of individual AD medications were converted into defined daily dose (DDD) units and subsequently pooled across all ADs to obtain the overall yearly AD prescription level. The AD prescription ratio was obtained by dividing this yearly AD prescription level by the overall volume of prescribed units for all reimbursed medications. Thus, the AD prescription ratio quantified the level of AD prescription in relative terms. This variable was analyzed both as a continuous variable and as a dichotomous variable, using the median as the threshold to define higher and lower prescribers.

### Statistical methods

The analysis explored the influence of all characteristics of GPs and their patients on the AD prescription ratio. We first analyzed the AD prescription ratio as a continuous variable. Univariate analyses relied on parametric (i.e., Student’s t-test or one-way ANOVA) or non-parametric (i.e., Mann-Whitney’s non-parametric test or Kruskal-Wallis test) methods depending on the distribution of the characteristic considered. Variables separately associated with the AD prescription ratio at the 0.05 level were retained for multivariate analysis. In multivariate analysis, variables independently associated with the AD prescription ratio at the 0.05 level were determined using multiple linear regression and backward stepwise selection. Second, the AD prescription ratio was analyzed as a dichotomous variable (see above), and the same general approach was used with appropriate methods. Namely, univariate analysis relied on Pearson’s chi-square test or Fisher’s exact test as appropriate and multivariate analysis relied on logistic regression and backward stepwise selection. Softwares Epi-info (version 3.5.3) and SAS (version 9.2, SAS Institute, Cary, North Carolina) were used.

## Results

### Description of the sample

The 816 GPs included in the study had a mean age of 52.7 years. Overall, they performed 5,186,461 consultations in a population of 1,248,443 inhabitants in 2010. On average, they saw a mean of 1,059 different patients over the year and performed 3,922 consultations and home visits. The overall density and medical activity of the GPs included in the study was similar to that for France as a whole. In 2010, there were 53,422 physicians registered as GPs in France, which has a population of 65 million. GPs in France had a mean of 4,319 consultations or home visits in 2010 [19,20].

### Characteristics of the AD prescription ratio

**Figure 2:**
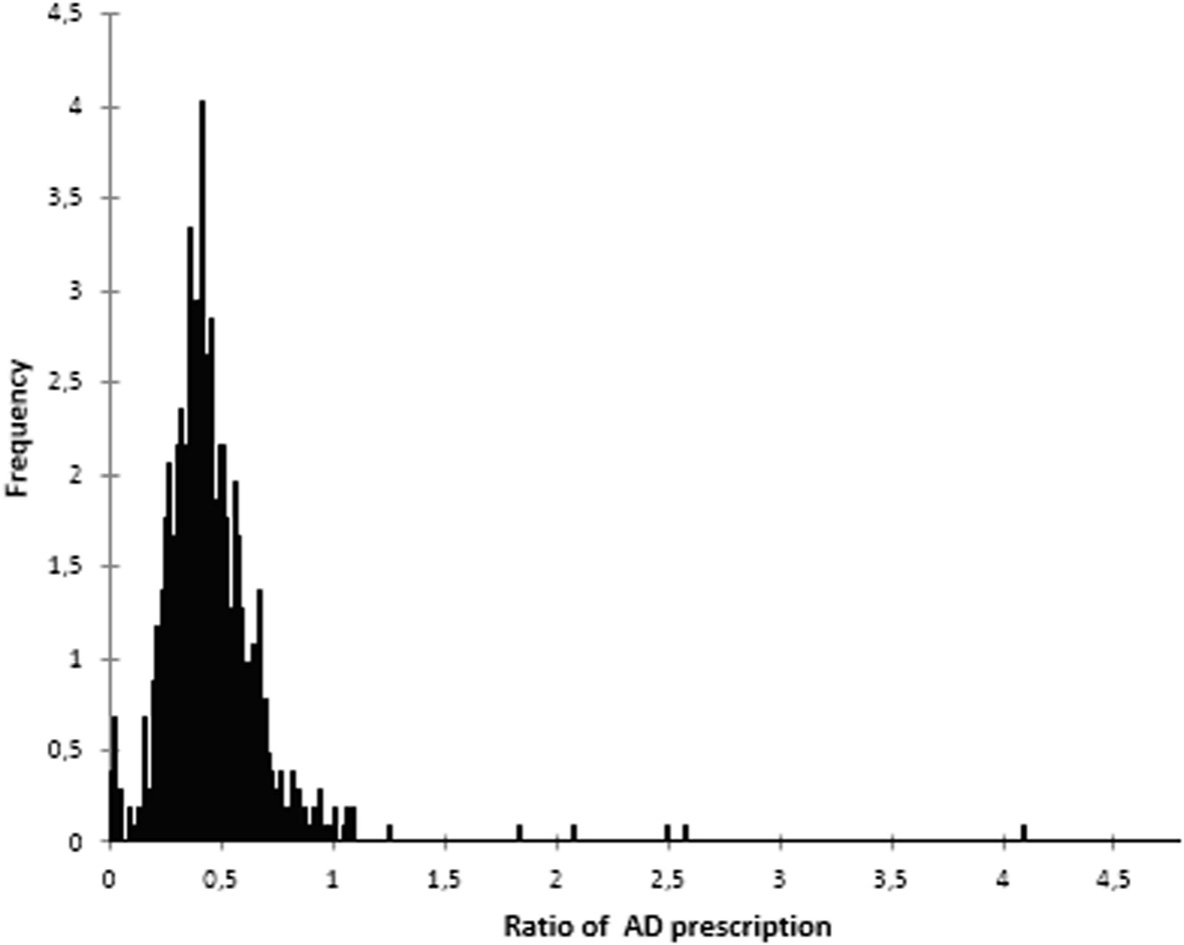
**Distribution of the ratio of AD prescription among the 816 GPs.**

The mean and median numbers of prescribed boxes (packaging sets) of all drugs were, respectively, 27,406 (standard deviation 15,972) and 26,421 (range 149 to 107,878). The mean and median numbers of prescribed ADs were, respectively, 12,005 DDD (standard deviation 7,841) and 11,019 (range 4 to 50,594). The mean and median AD prescription ratios were, respectively 0.45 (standard deviation 0.25) and 0.42 (range 0.004–4.00). The AD prescription ratio had a roughly symmetric distribution in the range 0–1 with a small peak for very low values (24 values were less than 0.1) and a few high values (4 values were more than 2) (Figure 2).

**Table 1:** **Characteristics of the study sample (** ***n*** **= 816)**

		**Low prescribers (** ***n*** **= 408)**			**High prescribers (** ***n*** **= 408)**		
		**Number**	**Percent**	**[95% confidence interval]**	**Number**	**Percent**	**[95% confidence interval]**
Gender	Female	116	28.4	[24.2–33.1]	118	28.9	[24.6–33.6]
	Male	292	71.6	[66.9–75.8]	290	71.1	[66.4–75.4]
Practice location	Rural	110	27.0	[22.8–31.6]	80	19.6	[15.9–23.9]
	Urban	298	73.0	[68.4–77.2]	328	80.4	[76.1–84.0]
GP partially particular mode of practice	Yes	40	9.8	[7.2–13.2]	13	3.2	[1.8–5.5]
	No	368	90.2	[86.9–92.9]	395	96.8	[94.5–98.2]
Cessation of activity during the year 2010	Yes	7	1.7	[0.8–3.7]	7	1.7	[0.8–3.7]
	No	401	98.3	[96.3–99.2]	401	98.3	[96.3–99.2]
		*Mean*		*[Standard deviation]*	*Mean*		*[Standard deviation]*
Age of GPs		52.6		[9.39]	52.8		[8.10]
Cumulative years of medical practice		19.83		[10.71]	20.5		[9.7146]
Number of consultations and home visits		3,982		[1,994]	3,863		[1,758]
Number of different patients per year		1,106		[538]	1,012		[396]
Number of registered patients		701		[406]	732		[346]
Mean age of patients		46.3		[6.02]	45		[6.1]
Number of patients with low income		60		[79]	45		[48]
Number of patients with chronic disease		145		[91]	144		[80]
Number of days of Sickness certificates prescribed		3,608		[2,537]	3,571		[2,225]
Number of patients prescribed a sickness certificate		100 [64]			94		[50]
Total volume of reimbursement (number of units)		28,247		[17,316]	26,565		[14,477]
Number of prescriptions orders		3,847 [2,122]			3,808		[1,859]
% ≥ 70 new AD		0.86		[0.34]	0.95		[0.20]
Number of DDD		9,163		[6298]	14,848		[8,200]
AD prescription ratio		0.30		[0.09]	0.60		[0.27]

### Characteristics of GPs and their patients (Table 1)

Among the 816 GPs, 234 (28.7%) were female and 582 male (71.3%). The less common category of GP’s age was the youngest (i.e., <40 years) age category (*n* = 72, 8.8%) and the most frequent was the 50–59-year category of age (*n* = 345, 42.3%). Overall, 190 GPs (23.3%) practiced in rural areas. On average, GPs had 716 registered patients with mean age 46 years and took care of 53 low-income patients and 145 patients with a registered chronic condition allowing for full coverage of medical expenses by their health insurance.

**Table 2:** **Characteristics of the study sample (** ***n*** **= 816) and results of univariate analysis for the AD prescription ratio as a continuous variable and as a dichotomous variable (<0.42 for “low” prescribers and ≥0.42 for “high” prescribers)**

		**Number**	**Percent**	**Mean AD prescription ratio [standard deviation]** ^**b**^	**Odds ratio (95% confidence interval)** ^**c**^
Characteristics of the GPs					
Gender	Female	234	28.7	0.47 [0.22]	1
	Male	582	71.3	0.44 [0.26]	0.98 (0,84;1.15)
Age category	<40 years	72	8.8	0.39 [0.15]	1
	40 to 49 years	198	24.3	0.47 [0.21]	1.58 (1.26;1.96)
	50 to 59 years	345	42.3	0.47 [0.24]	1.53 (1.26;1.86)
	≥60 years	201	24.6	0.43 [0.31]	1.21 (0.99;1.47)
Practice location	Rural	190	23.3	0.41 [0.16]	1
	Urban	626	76.7	0.46 [0.27]	1.20 (1.05;1.4)
GP partially particular mode of practice	Yes	53	6.5	0.39 [0.67]	0.63 (0.53; 0.7)
	No	763	93.5	0.45 [0.19]	1
Cessation of activity during the year 2010	Yes	14	1.7	0.46 [0.21]	0.84 (0.45;1.54)
	No	802	98.3	0.45 [0.25]	1
Cumulative years of medical practice	<10	155	19	0.42 [0.19]	1
	10–19	213	26.1	0.45 [0.20]	1.06 (0.87;1.30)
	20–29	267	32.7	0.47 [0.23]	1.18 (0.97;1.44)
	>30	181	22.2	0.46 [0.35]	1.04 (0.85;1.27)
Characteristics of the GP’s practice and patients in 2010					
Number of consultations and home visits	<1,999	113	13.8	0.51 [0.50]	1
	2,000 to 3,999	320	39.2	0.45 [0.22]	1.00 (0.80;1.24)
	4,000 to 5,999	296	36.3	0.44 [0.14]	1.03 (0.82;1.28)
	≥6,000	87	10.7	0.40 [0.15]	0.81 (0.63;1.05)
Number of different patients per year	<999	369	45.2	0.47 [0.31]	1
	>1,000	447	54.8	0.43 [0.18]	0.90 (0.79;1.04)
Number of registered patients	<499	213	26.1	0.46 [0.42]	1
	500–1,000	435	53.3	0.45 [0.15]	1.21 (1.04;1.41)
	>1,000	168	20.6	0.45 [0.15]	1.28 (1.05;1.57)
Mean age of patients	<40	83	10.2	0.46 [0.37]	1
	40–49	574	70.3	0.46 [0.20]	1.11 (0.89;1.38)
	≥50	159	19.5	0.43 [0.34]	0.92 (0.72;1.18)
Number of patients with low income*	<39	478	58.6	0.46 [0.30]	1
	40–79	176	21.6	0.45 [0.16]	0.95 (0.80;1.14)
	≥80	162	19.9	0.41 [0.14]	0.77 (0.66;0.90)
Number of patients with chronic disease^a^	<100	244	29.9	0.48 [0.40]	1
	100–199	361	44.2	0.45 [0.14]	1.06 (0.86;1.25)
	200–299	175	21.4	0.43 [0.14]	0.93 (0.77;1.12)
	≥300	36	4.4	0.40 [0.10]	0.90 (0.66;1.24)
Number of days of sickness certificates prescribed	<1,999	223	27.3	0.46 [0.42]	1
	2,000–3,999	281	34.4	0.46 [0.14]	1.34 (1.12;1.60)
	4,000–5,999	191	23.4	0.44 [0.15]	1.12 (0.93;1.38)
	≥6,000	121	14.8	0.42 [0.14]	1.00 (0.82;1.22)
Number of patients prescribed a sickness certificate	<100	454	55.6	0.46 [0.31]	1
	≥100	362	44.4	0.44 [0.14]	0.97 (0.84;1.11)
Prescription of new ADs	≥70% new AD	741	90.8	0.46 [0.25]	1.60 (1.38;1.86)
	<70% new ADs	75	9.2	0.33 [0.17]	1
Total volume of reimbursement (number of units)	0–19,999	261	32	0.49 [0.39]	1
	20,000–39,999	397	48.7	0.44 [0.14]	0.96 (0.82;1.13)
	≥40,000	158	19.4	0.41 [0.15]	0.87 (0.75;1.01)
Number of prescriptions orders	0 to 1,999	152	18.6	0.49 [0.49]	1
	2,000 to 3,999	298	36.5	0.45 [0.16]	1.11 (0.92;1.35)
	4,000 to 5,999	270	29.9	0.44 [0.14]	1.08 (0.89;1.31)
	≥6,000	96	9.7	0.42 [0.13]	0.98 (0.77;1.24)

*Patients with low income, treated free of charge, with no direct payment to the GP.

^a^Chronic diseases, at least one of 30 chronic conditions such as type 2 diabetes, or chronic heart failure.

^b^AD prescription ratio as a continuous variable.

^c^Relative to the dichotomized AD prescription ratio using the lower prescription level as the reference level.

### Predictors of AD prescription—univariate analysis (Table 2)

The AD prescription ratio as a continuous variable was statistically significantly higher in relation with greater GP’s age (*p* = 0.008), urban location (*p* = 0.01), larger overall volume of reimbursement (*p* < 0.001), larger number of registered patients (*p* = 0.02), higher prescription of new ADs (*p* = 0.001), larger number of patients being prescribed sickness certificate days (*p* = 0.001), higher overall volume of reimbursement (*p* = 0.01), larger number of different patients seen per year (*p* = 0.001), larger overall number of patients seen (*p* = 0.04), larger number of low-income patients (*p* = 0.006), and larger number of patients with a chronic disease (*p* = 0.02).

After dichotomizing the AD prescription ratio, physicians between 40 and 59 years of age prescribed more ADs compared to younger ones. Physicians working in urban areas were higher AD prescribers than those working in rural areas. Those with more than 500 registered patients were higher AD prescribers than those with fewer registered patients. A higher number of sickness certificates were associated with a higher AD prescription rate. GPs prescribing new ADs frequently (>70% of all ADs prescribed) were also higher AD prescribers. Conversely, GPs with more low-income patients prescribed fewer ADs.

**Table 3:** **Multivariate analysis: variables independently associated with the dichotomized AD prescription ratio (<0.42 for lower prescribers and ≥0.42 for higher prescribers)**

**Variable**	**Mean difference in AD prescription ratio [95% CI]** ^**b**^	**OR [95% CI]** ^**c**^
GP’s age		
**≥**60	0	1
50–59	0.026 [−0.023; 0.074]	1.87 [1.29; 2.71]
40–49	0.038 [−0.049; 0.080]	1.74 [1.14; 2.64]
<40	−0.068 [−0.14; 0.00]	0.65 [0.35; 1.2]
Practice location:		
Rural	0	1
Urban	0.056 [0.016; 0.096]	1.954 [1.3; −2.80]
Number of consultations/year/GP*		0.93^a^ [0.90–0.97]
Number of patients with low income		
≥80		1
40–79		1.69 [1.06; 2.67]
<39		2.23 [1.45; 3.40]
Class of prescription		
<70% new ADs	0	1
>70% new ADs	0.12 [0.058; 0.18]	3.45 [1.94; 6.15]
Cumulative number of sick leave days prescribed per year		
**≥**6,000		1
4,000–5,999		0.91 [0.55; 1.47]
2,000–3,999		1.12 [0.68; 1.84]
Fewer than 1,999		0.43 [0.24; 0.78]
Total volume of drugs reimbursed	−0.0053* [−0.0084; −0.00233]	
Total volume of prescription orders	0.0462** [0.0184; 0.0740]	
Number of consultations and home visits	−0.0128*** [−0.0179; −0.0077]	

*Mean difference per 1,000 additional drugs reimbursed (quantitative variable).

**Mean difference per 1,000 additional prescription orders (quantitative variable).

***Mean difference per 100 additional consultations or home visits.

^a^Odds ratio per 100 additional consultations (quantitative variable).

^b^From multiple linear regression applied to the AD prescription ratio as a continuous variable.

^c^From multiple logistic regression applied to the dichotomized AD prescription ratio using the lower prescription level as the reference level.

### Predictors of AD prescription—multivariate analysis (Table 3)

In multivariate analysis, upon considering the AD prescription ratio as continuous, the factors independently showing a positive association with higher AD prescription were intermediate GP’s age (40 to 59 years) and urban practice location, whereas younger GP’s age (<40 years) was associated with lower AD prescription. For instance, GPs with a practice in an urban area had a higher AD prescription ratio than GPs practicing in rural areas, with a mean difference of 0.056 (95% confidence interval 0.016 to 0.096). Regarding prescription and overall activity, higher total volume of drugs reimbursed and higher number of consultations and home visits were independently associated with lower AD prescription (i.e., inverse association). Finally, GPs prescribing new ADs more frequently were also higher AD prescribers.

Upon considering the AD prescription ratio as dichotomous, analogous results were obtained for GP’s age and practice location. For instance, the odds ratio of higher AD prescription was 1.95 (95% confidence interval 1.36 to 2.80) for GPs practicing in an urban area relative to GPs practicing in a rural area. A higher number of low-income patients in the practice were inversely associated with AD prescription. Regarding prescription and overall activity, number of consultations and number of sick leave days prescribed were inversely associated with AD prescription. Finally, GPs prescribing new ADs more frequently were again higher AD prescribers.

## Discussion

From this study, it appears that the profile of the “higher” prescriber was an urban, middle-aged GP (40–59 years of age), prescribing a higher proportion of new ADs and seeing fewer low-income patients. Moreover, lower medical activity might be added to this typical profile since the variables related to medical activity (i.e., overall volume of reimbursement, number of prescriptions per year, number of different patients seen per year, number of consultations and home visits, number of sick leave days prescribed) tended to be inversely associated with AD prescription. This suggests that GPs might have different prescription patterns depending on their practice burden.

Some data showed that women practiced differently and tended to take social problems more into account [21]. In our study, however, there was no gender effect.

The main indications for prescribing ADs are depression and psychological problems [22]. France has the highest proportion of patients taking ADs in Europe, but because of shorter periods of use, France finished only in the middle of the pack in a ranking of European countries for total AD prescriptions [23]. Possible over-prescription of ADs as well as a “drug prescription culture” has been highlighted in France [24]. The figures from our study seem to contradict the idea that high workload is related to over-prescription of ADs, due to incorrect assessment of patients.

### Strengths and limitations

No *a priori* sample size calculations were performed, but our study was conducted on a large sample, so that statistical power was high. Only the database of the CNAM-TS was used in this study but this represents 80% of the population. Moreover, after having applied selection criteria, our sample was almost exhaustive. The average number of consultations per GP in our sample (*n* = 3,922) was close to that in French GPs (*n* = 4,319). The influence of PMPs in the univariate analysis underlines the fact that excluding those who had a large share of partial PMPs was relevant.

In the absence of a universally recognized measure of AD prescription level, the AD prescription ratio was devised for this study. We chose a relative measure of AD prescription in order to eliminate the influence of overall prescription level. Moreover, the numerator was expressed in DDD in order to have an accurate quantification of AD prescription. It was not possible to express the denominator in terms of DDD as well since the CNAM-TS database used in this analysis does not include information on indication. Thus, for medications with several possible indications (e.g., non-steroidal anti-inflammatory drugs or pain killers), a unique DDD calculation was not possible. This is why the denominator was expressed in terms of medication units, yielding a hybrid ratio. Alternatively, an absolute measure of AD prescription could have been used. For instance, using the same numerator but using the number of patients seen in the year 2010 as the denominator would yield a rate of AD prescription per patient. This absolute AD prescription rate and our AD prescription ratio have different (albeit correlated) denominators and do not capture the same AD prescription features. For instance, a low AD prescription rate may correspond to either a low AD prescription ratio if the GP is a low AD prescriber but an average-to-high drug prescriber overall or a high AD prescription ratio if the GP is a low drug prescriber overall and an even lower AD prescriber, two very different situations that can be distinguished upon using the AD prescription ratio but not the AD prescription rate. Thus, results could be different with the AD prescription rate compared to those obtained with the AD prescription ratio, and our results have to be interpreted with this element of caution in mind.

Since only variables present in the database were used, it is possible that important determinants of AD prescription were missed such as detailed individual patient characteristics. Our study relied on an overall assessment of GPs’ AD prescriptions. We do not have figures on referral for diagnosis and treatment. A prescription renewed by a GP could have initially come from secondary care.

As well, AD prescription is a proxy to identify depression and psychological distress, but ADs are also prescribed for non-psychiatric reasons, though the exact proportion of this phenomenon is unknown [10,25,26]. This impacts the relevance of our conclusions on GP behavior. Finally, our study did not address the overall usefulness and appropriateness of prescriptions since the database that we used does not include indications. This also means that researchers and policymakers need to interpret prescription levels in a careful manner.

### Hypotheses and further implications

Middle-aged GPs (40–59 years of age) seemed to prescribe ADs more than younger or older GPs. However, the number of years of practice, partially related to a physician’s age, seemed to have no influence on AD prescription. Prescribing “old” ADs was independently related with a lower ratio of AD prescription. Rural GPs prescribed ADs less than did urban practitioners. As data on precise assessment of the patients’ conditions were lacking, only hypotheses can be made: for example, elderly patients are more likely to have an older GP and to be depressed [25]. The sensitivity and odds of diagnosis of depression by older non-psychiatric physicians could be lower [26]. However, older GP’s age could also be related to greater experience and a better understanding of the patient, thus avoiding over-prescription of ADs. Differences between rural and urban areas may also be explained by over-prescription in urban areas, but the lack of higher AD prescriptions among “at risk” populations, such as low-income people, makes this explanation less likely.

Due to differences in the sample characteristics, and in the variables assessed, our data cannot be directly compared with those of the study of Morrison on the same subject [27]. Nevertheless, conclusions concerning the association of urban practices with greater AD prescription are similar. Conversely, our results do not show any influence of risk factors for depression, which may suggest a lack of diagnosis recognition, or other prescriptions other than ADs, such as psychological care. The conclusions on the influence of GP’s age are not consistent with those of Morrison either, with the oldest GPs prescribing less than middle-aged GPs in our study. In either study, findings do not indicate whether there is a lack of recognition of depression by older GPs or a more easy prescription for moderate depression among younger GPs.

Although chronic diseases and low income are well-known risk factors for depression [25-28], only the latter was found associated with increased AD prescription with an inverse association in our study. Rural patients received fewer AD prescriptions, although we know depression also occurs in rural settings [28]. This might be related to under-detection in this context. It is known that all GPs tend to have difficulties in recognizing patients’ needs for mental care. Structural heath care system problems, such as difficulties in accessing mental health care services, have already been pointed out [29-31]. Poor coordination between secondary and primary care has also been highlighted [30,32]. Better coordinated care could improve prescription quality outcomes [33,34]. Enhancing shared decisions and shared care has been shown to be effective [33,35]. AD prescriptions have to be assessed on relevant outcomes such as increased prescription among chronic patients with comorbidities and those who belong from lower social classes.

To go further and understand individual GPs’ behavior would require a more in-depth assessment of the patients’ situations including all biopsychosocial components of the medical situation. But, we also know that GPs AD prescription behavior relies on many personal and contextual factors [36], such as adherence to the guidelines, availability of psychotherapists, and beliefs on ADs’ efficacy and effectiveness. Other influences such as prior training or experience of prescribing antidepressants also have to be taken into account in future studies, to assess the way this impacts GPs’ prescription strategies.

## Conclusions

Our study found that the profile of the higher prescriber was an urban, middle-aged GP (40–59 years of age) more often prescribing recent ADs and having fewer low-income patients. The total volume of reimbursement and the volume of consultations did not increase the prescription ratio. These findings do not support the idea that workload is the cause of over-prescription of ADs.

Further understanding would require analyzing both the influence of patients’ situations and other GPs’ characteristics, such as the use of guidelines, prior training, or prior experience of prescribing ADs. In-depth assessment of all biopsychosocial components of the medical situation and other influences on GPs behavior would be useful to better explain AD prescriptions.

## Additional file

Additional file 1:
**Table ADs included in the analysis.**

## Abbreviations

AD: Antidepressants
GP: General practitioner
DDD: Defined daily dose
CNAM-TS: Main health insurance system in France
PMP: Particular mode of practice
INSEE: National Institute of Statistics and Economic Studies
