# Supplementary material for: How do GP practices and patient characteristics influence the prescription of antidepressants? A cross-sectional study
Source: Ann Gen Psychiatry. 2015 Jan 22;14:3. doi: 10.1186/s12991-015-0041-7 (PMC4308843; doi:10.1186/s12991-015-0041-7)
Supplement: Additional file 1: — Table ADs included in the analysis. [file 12991_2015_41_MOESM1_ESM.docx]

**Additional file 1 - table ADs included in the analysis**

| « Old  Molecules » | Tricyclic agents | Amoxapine  Amitriptyline  Clomipramine  Dosulépine  Doxépine  Imipramine  Maprotiline  Trimipramine |
| --- | --- | --- |
|  | MAOIs | Marsilid  Moclamine |
| « New molecules » | SSRIs | Citalopram  Escitalopram  Fluoxétine  Fluvoxamine  Paroxétine  Sertraline |
|  | SNRIs | Duloxétine  Milnacipran  Venlafaxine |
|  | Non MAOI non TCA agents | Miansérine  Mirtazapine  Tianeptine |
